# Search for Alternative Control Strategies of *Drosophila suzukii* (Diptera: Drosophilidae): Laboratory Assays Using Volatile Natural Plant Compounds

**DOI:** 10.3390/insects11110811

**Published:** 2020-11-18

**Authors:** Astrid Eben, Frank Sporer, Heidrun Vogt, Pille Wetterauer, Michael Wink

**Affiliations:** 1 Julius Kühn-Institute (JKI), Federal Research Centre for Cultivated Plants, Institute for Plant Protection in Fruit Crops and Viticulture, Schwabenheimer Straße 101, 69221 Dossenheim, Germany; heidrun.vogt@julius-kuehn.de; 2 Institute for Pharmacy and Molecular Biotechnology (IPMB), Im Neuenheimer Feld 364, Heidelberg University, 69210 Heidelberg, Germany; frank.sporer@gmx.de (F.S.); p.wetterauer@uni-heidelberg.de (P.W.); wink@uni-heidelberg.de (M.W.)

**Keywords:** capillary feeding, contact toxicity, essential oils, oviposition, natural plant products, spotted wing drosophila

## Abstract

**Simple Summary:**

Adult flies of the invasive fruit pest, *Drosophila suzukii*, commonly known as spotted wing drosophila, showed susceptibility towards several natural plant products tested in bioassays under laboratory conditions. Depending on the compound tested, contact toxicity, modified food uptake, or reduction in numbers of eggs deposited and hatched was found. The natural plant substances thereby identified will be further assessed under field conditions and can be used to develop innovative pest control strategies.

**Abstract:**

*Drosophila suzukii* (Diptera: Drosophilidae), is native to southeastern Asia and invaded Europe during the past decade. It causes serious economic damage in cherries and soft fruits. Control strategies rely on few insecticides with varying success. Due to environmental concern, the use of synthetic chemicals is restricted. Therefore, research effort is put into the quest for alternative substances applicable in chemical pest control. In laboratory assays, we tested 17 volatile plant compounds from different chemical classes for their contact toxicity, feeding modification, and oviposition repellency. Toxicity through contact with treated surfaces was evaluated after 1 h, 4 h, and 24 h; effects on food uptake were observed with capillary feeding (CAFE)—tests and oviposition trials compared egg numbers laid in raspberry medium with or without treated filter paper. Cinnamon oil and its components had the highest contact toxicity with an LC_90_ = 2–3%, whereas lemongrass oil, its main components, and farnesol were less toxic (LC_90_ = 7–9%), and geraniol was the least toxic. In CAFE tests, feeding stimulation was observed through 0.1% and 1% solutions of citronellol, lemongrass oil and farnesol. Cinnamon oil, cinnamaldhyde, and ethyl cinnamate were not consumed at a concentration of 1%. In the presence of citral, eugenol, and lemongrass oil, oviposition was reduced, and in the presence of limonene, no eggs were deposited. The natural products found most efficient in either bioassay will be further tested under field conditions.

## 1. Introduction

The invasive spotted wing drosophila (SWD), *Drosophila suzukii*, has become the most important worldwide pest species in cherries and many soft berry fruits within less than a decade of its first appearance [1,2,3,4,5,6]. It causes high economic damage due to its polyphagous feeding habit with over 80 known hosts, as well as its rapid generation turn-over, with more than eight generations per year [4]. Population dynamics are largely influenced by annually variable weather conditions [7,8,9,10,11]. Thus, outbreak prognostics are difficult, vary among regions, and often differ greatly for subsequent years [12].

SWD females have a serrated, hardened, and enlarged ovipositor that allows oviposition in healthy, undamaged, and ripe fruits [13]. SWD larvae develop inside the infested fruits, which makes these fruits collapse and unmarketable. Under favorable climatic conditions, the flies’ development from egg to adult takes from 10 to 17 days [7]. Such a short generation time and the infestation of ripening and ripe fruits require repeated insecticide treatments as well as applications close to harvest. This situation poses large difficulties to integrated pest management (IPM) strategies. Resistance development through repeated application is another serious threat [14]. Currently used efficacious insecticides are problematic due to their potential harmful side effects on beneficial insects and pollinators [15,16]. Moreover, residue limits on harvested fruits and pre-harvest intervals are a main concern for producers. Sanitary measures, such as removal of dropped fruits and ground covering vegetation, pruning of fruit bearing plants in order to keep the plants exposed to sunlight, and reduction in humid, dark hiding places, are therefore of key importance [17,18].

To date, insect exclusion netting has been found to be a sustainable control strategy [19,20,21]. Research is ongoing to identify fly resistant or less susceptible cultivars for the most important commercial fruits. Nevertheless, laboratory studies of fruit susceptibility were not able to clearly identify parameters (e.g., skin penetration force, sugar content, color, fruit volatile composition) influencing the preferences of egg-laying females [22,23,24,25]. Studies of endemic natural enemies for biological control of *D. suzukii* in the invaded areas have identified pupal parasitoid species of the Hymenoptera [26,27], *Trichopria drosophilae* (Perkins) (Pteromalidae) and *Pachycrepoideus vindemmiae* (Rondani) (Diapriidae) as most promising candidates for commercial production and release. Laboratory and field trials confirmed this for Switzerland [28] and Germany [29,30]. However, because both species parasitize only the pupal stage of the spotted wing drosophila, economic damage is still incurred. Pupal parasitism can reduce the number of adult flies that build up the next generation and is thus considered an important tool used in combination with other means of control. One Asian specialist parasitoid for possible classical biological control has been identified as well, the larval parasitoid *Ganaspis brasiliensis* (Figitidae). Detailed studies are ongoing before potential release can be discussed [27].

Since the beginning of its invasive history, projects were conducted with the goal to identify attractive volatile compounds for adult SWD from its host plants, its host fruits, and from fruit-associated yeast species [31,32,33,34,35,36]. Those substances might potentially serve as species specific lures in traps and for monitoring of the flies [37,38,39,40,41,42,43,44,45,46]. Plant secondary metabolites (i.e., azadirachtin and pyrethrum) and essential oils (EOs) are commonly used biopesticides [47,48,49,50,51,52,53,54]. Some of these natural plant products [55,56,57,58,59,60], and volatile compounds from host fruits [61,62,63], were tested on different life stages of SWD [55,56,64]. In laboratory studies, a number of promising plant-derived substances have been identified [61,62,65,66]. Nevertheless, the use of these compounds in monitoring traps under field conditions showed that these lures were not sufficiently species specific [41,42,67]. Despite the intensive search, to date no single plant-based substance has been identified as primary candidate for efficient control strategies [23,24,68]. Moreover, since each female fly can deposit numerous eggs in one fruit, and infested fruits collapse within days due to larval feeding, it is most urgent for efficacious control strategies to find a suitable repellent substance [59,69].

The objective of our laboratory study was to find candidate volatile natural plant products for environmentally sustainable and behavior-based control strategies against SWD based on toxicity and behavior-modifying effects.

## 2. Materials and Methods

### 2.1. Insect Rearing

SWD flies were reared and kept under controlled conditions of 23 °C, 60% RH, and a photoperiod of 16 h:8 h light:dark cycle in a climate chamber at the JKI in Dossenheim (Heraeus Vötsch, Industrietechnik GmbH, Germany). The colony was maintained on the JKI standard diet: 30 g sugar (Südzucker, Mannheim, Germany) 142 g cornmeal (Frießinger Mühle GmbH, Bad Wimpfen, Germany), 20 g soy flour (Schoenenberger GmbH, Magstadt, Germany), 34 g brewer’s yeast (Diana, Diekmann GmbH, Versmold, Germany), 11.2 g agar (Roth GmbH, Karlsruhe, Germany), 5 g vitamin mixture (MP Biomedicals™ Vanderzant, Fisher Scientific, Schwerte, Germany), 9.4 mL propionic acid (Roth GmbH), 1748 mL water, modified from a recipe by Fondazione Edmund Mach, San Michele, Italy. For oviposition assays, this same medium was offered in 50 mL containers (Huhtamäki, Espoo, Finland) and replaced every 48 h. The containers were stored in the rearing chamber until adult eclosion. Newly eclosed flies were grouped in cages with a maximal age difference of 3 d. A mix of dried brewer’s yeast and household sugar 1:1, plus a 5% sugar/water solution, served as additional food supply for the adult flies. The flies used in all bioassays were 7–10 d old and had previous mating and oviposition experience.

### 2.2. Chemical Substances

Seventeen natural plant products were chosen for the assays. All compounds used were present in liquid form (Table 1). The test substances included terpenoids, essential oils and some of their respective major components. They were chosen based partly on their common presence in the head space of cultivated plants (benzaldehyde, geraniol, caryophyllene), as components of essential oils found in seeds of various edible fruits (farnesol, phellandrene) [70] or because of their known deterrent effects against pest insects (limonene, lemon grass oil) [71]. Star anise oil, cinnamon oil, lemongrass oil, and citral are broadly used in human food items [72].

### 2.3. Bioassays

To test the compounds for their potential toxicity towards adult SWD through direct contact, feeding stimulation or repellency, and potential modification of oviposition behavior, the selected natural compounds were screened using three different bioassays. Test insects were removed from the colony with an aspirator. A disposable polyethylene pipette attached to the aspirator served as recipient for each of the fly groups before their placement into the test unit. Prior to fly release in the respective test arena or test vial, the pipettes containing the flies were briefly (<30 s) cooled on ice to prevent their escape.

#### 2.3.1. Contact Toxicity

Test compounds were dissolved in a 5% lecithin–water solution. Preliminary screening at the highest concentration of 10% revealed no negative impact on adult SWD under the test conditions for lavender oil, limonene, and phellandrene. These substances were not further tested. All other substances were tested for contact toxicity with adult SWD at 0.1%, 0.5%, 1%, 2.5%, and 5%. The tests revealed an estimated LC_50_ around 3–6%. Thus, for the final assays we used the following five concentrations for each test substance: 2%, 4%, 6%, 8%, and 10% (i.e., 200 µL test substance in 2 mL applied solution). Transparent polystyrol containers (50 mL, Huhtamäki, Finland) were used as experimental units. Test substances were sprayed with a spray finger onto the bottom and sides of the test containers. A circular opening was cut in the lid and covered with fine gauze to avoid saturation with the test volatiles. The surface coverage obtained was 1.5 mg/cm^2^. Prior to release of the test insects, the spray cover in the containers was allowed to evaporate to dryness. Ten flies were then placed simultaneously in each container as groups of five males and five females. All concentrations of each substance were tested in six replicates (n = 6). Test containers were kept under a fume hood (23 °C, 16 h:8 h light:dark cycle, 500 m^3^/h). Control cups were sprayed with lecithin–water solution (5%). Mortality through contact with the treated surface was evaluated visually after an exposure time of 1 h, 4 h, and 24 h for the flies in each container.

#### 2.3.2. Capillary Feeding

**Figure 1:**
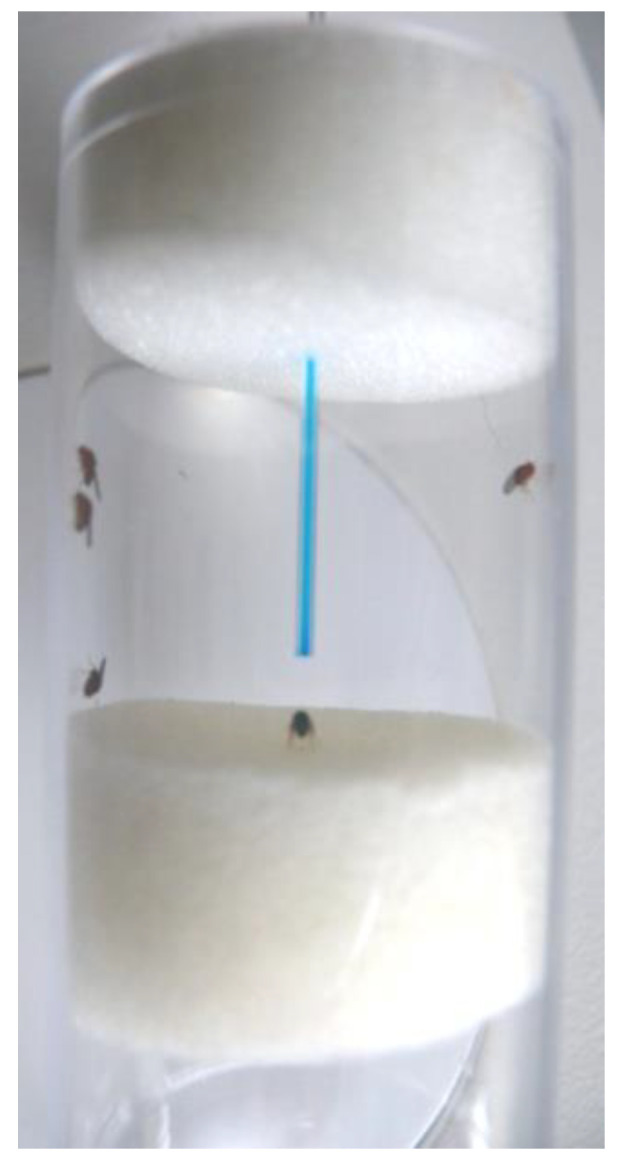
*Drosophila* vial used in capillary feeding (CAFE) tests with inserted glass capillary containing test liquid and five female *D. suzukii* flies (Photo: J. Just, JKI).

A modified capillary feeding test (CAFE) was conducted to detect repellent or stimulatory effects through feeding on liquid natural compounds [73]. Preliminary assays showed that female flies were more responsive then males, therefore only females were used for further assays. Test females were provided only with sugar-water (5%) for 20 h prior to the assays. All natural compounds were offered in three concentrations (0.01%, 0.1%, 1.0%) as emulsions with brewer’s yeast (0.5%), fructose (2%), and lecithin (soy lecithin, 97.5% as phospholipids, Caelo, Hilden, Germany). From each test substance, 6 µL test or control emulsion was offered in 20 µL disposable glass capillaries (Brand^®^, Sigma Aldrich, Germany). Brilliant blue (E133, FD&C, Pharmorgana GmbH, Eppstein, Germany) was added (0.5%) in order to be able to distinguish individual flies that consumed the offered liquid. The abdomen of these females turned into a visible blue color through uptake of the test substances from the capillaries. During the 4 h test period, Drosophila vials (VWR, Bruchsal, Germany) with five test females each were kept under controlled conditions in climate chambers (23 °C, 60–70% RH; Rumed^®^, Rubarth Apparate Company, Laatzen, Germany) (Figure 1). Vials without flies served as controls to detect loss of the test emulsion through evaporation. An emulsion without the test substance served as control. After 4 h, all flies were removed from the test vials and dead flies were counted. Only the number of flies with a blue abdomen, which indicated feeding, were recorded for each of the assay vials. After 4 h, the remaining amount of the tested substance in each glass capillary was measured. For each capillary, the consumption within 4 h was calculated by subtracting the mean loss through evaporation in vials without flies from the recorded remaining amount of the test solution after feeding. All substances and concentrations were tested with ten replications (n = 10).

#### 2.3.3. Oviposition Assay

A transparent agar-medium (1.5% Agar-Agar) colored red through the addition of 2.5% raspberry juice concentrate (ADM Wild Ingredients GmbH, Heidelberg, Germany) was used as oviposition substrate. Five milliliters of the oviposition medium was filled in a polystyrol petri dish (5 cm diameter, Greiner Bio-One, Frickenhausen, Germany). Filter paper (VWR, Bruchsal, Germany) circles with ø 0.5 cm were cut with a paper punch. Five µL of the undiluted test substance was applied with a syringe onto the filter paper. Paper circles with 5 µL tap water served as control. Paper circles were left to dry for 15 min, then one filter paper circle was placed in the middle of a petri dish with oviposition medium. Petri dishes were transferred to a custom made, dismountable test arena made of a transparent plexiglass ring (10 cm diameter, 3 cm height) enclosed by glass plates (12 cm × 12 cm × 5 mm) on top and bottom [74]. Three mesh-covered openings in the plexiglass ring provided ventilation. The air in each test arena was constantly exchanged with the help of two aquarium pumps (38 mL/min) to avoid accumulation of volatiles from test substances. Each glass arena was individually connected through a fourth opening with a Teflon tube to the pump. The air was replaced with ambient air at room temperature from outside the walk-in-climate chamber (23 °C, 60–70% RH) (Heraeus Vötsch Industrietechnik GmbH, Germany). Five female flies (age: 7–10 d) were released into each test arena. During the first 60 min of the 3 h test period, the position of the five test females in the glass arena was recorded every 5 min. Location of a fly on either the oviposition medium, the petri dish or on the arena (glass plate or plexiglass ring) was annotated. After 3 h, all flies were removed and the number of eggs laid in each substrate was counted under a stereo microscope (Wild Heerbrugg AG, Switzerland). The number of dead flies in each arena was recorded. Oviposition media were kept at room temperature in the dark. After 26 h, the number of hatched larvae was counted for each test and control medium. All substances and controls were examined with eight replications (n = 8).

### 2.4. Chemical Analyses

Chemical stability of test solutions from CAFE assays was verified within 24 h after the assays. Samples were analyzed with a gas chromatograph (5890 Series II Hewlett Packard, Böblingen, Germany) coupled with a mass spectrometer (SSQ 7000 Finnigan MAT, Mascom, Bremen, Germany). A low polarity Zebron ZB-5 capillary column (30 m length, 0.25 mm inner diameter, 0.25 μm film thickness) was used for sample separation (Phenomenex, Aschaffenburg, Germany). Injection of samples (1 μL) was operated in split mode using helium as carrier gas (99.99% purity, Air Liquide, Düsseldorf, Germany). The temperature program began with an isothermal step at 40 °C for 2 min; temperature was increased at a rate of 6 °C/min up to 300 °C, and held constant for 5 min. The quadrupole mass detector was operated in electron-impact (EI) mode at −70 eV, 200 μA filament emission current, and 175 °C ion source temperature with 5 min filament delay time. Mass spectra were obtained at 40–500 m/z and recorded with Xcalibur^®^ 1.3 software (Thermo Fischer Scientific, Bremen, Germany). Peak retention time was compared with known standards from our local database. Volatile compounds from the samples were identified comparing the fragmentation pattern of their mass spectra with examples from previously analyzed authentic standards, and the Chemical Abstract Service database.

### 2.5. Statistical Analyses

The statistical analysis was performed with R version 3.6.3 [75] and the DescTools package [76]. For the contact toxicity test, the dose–response analysis was performed using the package drc [77]; the LC_50_ and LC_90_ values were estimated from a two-parameter log-logistic model for each treatment. The comparisons were conducted using the multcomp package [78]. For the capillary feeding test, a one-way ANOVA with Welch correction and a post-hoc Dunnett’s test were performed to study the significance of the mean differences from the untreated control for each treatment. For the oviposition assay, a one-sided *t*-test with Welch correction was performed to test the hypothesis that the treated flies lay less eggs than the control. The significance level in all tests was α = 0.05. Figures were prepared with the package ggplot2 [79].

## 3. Results

### 3.1. Bioassays

#### 3.1.1. Contact Toxicity

The bioassays evaluated toxicity of plant compounds to SWD through contact with treated surfaces. Benzaldehyde, citronellal, lavender oil, limonene, and phellandrene caused no mortality in preliminary screening assays at the highest concentration of 10% and were not further tested. Pure cinnamon oil was less toxic than some of its individual compounds: cinnamaldehyde, cinnamyl alcohol, ethyl cinnamate, and isoeugenol were highly toxic for SWD. Mortality was elevated even at the lowest tested concentrations (LC_50_ < 2%, LC_90_ < 3%). Lemongrass oil, citral, geraniol, and farnesol were less toxic (LC_90_ = 8–10%). Star anise oil and citronellol caused no mortality in the tested concentrations (LC_90_ > 10%) (Table 2). No flies died through contact with the lecithin–water-treated control cups after 1 h, 4 h, or 24 h.

Overall, males were more susceptible than females. We analyzed mortality over time since for several test compounds the comparison of mortality of male and female flies differed after 1 h, 4 h, or 24 h. We found a significant interaction between treatment and sex for cinnamaldehyde, cinnamyl alcohol, cinnamon oil, citral, and farnesol (Table 3). No significant differences in susceptibility to the tested substances between sexes were observed for citronellol, ethyl cinnamate, eugenol, geraniol, isoeugenol, lemongrass oil, and star anise oil.

After contact with cinnamyl alcohol, more males died after 1 h (*z* = −3.33, *p* = 0.002), for cinnamon oil after 4 h (*z* = −2.27, *p* = 0.043), for citral after 24 h (*z* = −4.31, *p* < 0.001). The contact with farnesol resulted in significantly higher mortality of male SWD only after 4 h (*z* = −5.97, *p* < 0.001) and 24 h (*z* = −4.65, *p* < 0.001), but not after 1 h of contact. Cinnamaldehyde significantly increased the mortality for all timepoints (after 1 h: *z* = −3.34, *p* = 0.002; after 4 h: *z* = −4.71, *p* < 0.001; after 24 h: *z* = −4.08, *p* = 0.0001).

#### 3.1.2. Capillary Feeding Test

**Figure 2:**
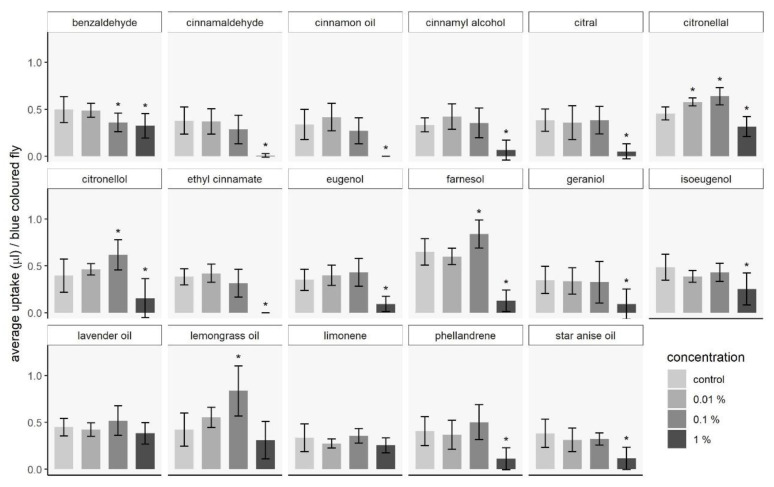
CAFE tests compared the uptake of the offered test solution per blue-colored fly for each of the three test concentrations and the control. Five females were released in each of the ten test vials per test substance concentration and control. Bars represent the mean value in µL consumed per fly during 4 h and corrected by the mean evaporated amount. Error bars show the standard deviation. Data were analyzed by one-way ANOVA and Dunnett’s *t*-test to significance level α = 0.05. Asterisks mark significant differences between the mean amount consumed from test substance and control.

The assessment of feeding stimulation or deterrence by test compounds offered in three different concentrations revealed variable responses shown as the mean number of flies with a blue-colored abdomen (Table 4). The blue dye used in our CAFE tests made visible that all substances were consumed by at least 50% (cinnamaldehyde) until up to 100% (citronellal) of the test females in concentrations of 0.01 and 0.1%. Lavender oil and limonene were consumed in the same amounts as the control liquid irrelevant of test substance concentration. Uptake was highest for lavender oil at any concentration (94%, 94%, 86%, respectively). Citronellal induced feeding at 0.01% (*t_dunnett_* = 18.18, *p* = 0.006), and 0.1% (*t_dunnett_* = 27.42, *p* < 0.001), whereas citronellol, lemongrass oil, and farnesol stimulated consumption at 0.1% compared to the lower concentration of 0.01% and the control (*t_dunnett_* = 8.57, *p* = 0.01; *t_dunnett_* = 10.76, *p* < 0.001; *t_dunnett_* = 12.35, *p* = 0.004, respectively). Fourteen out of seventeen test compounds significantly reduced feeding at the highest concentration of 1.0% (benzaldehyde: *t_dunnett_* = −13.46, *p* = 0.005, cinnamaldehyde: *t_dunnett_* = −23.48, *p* < 0.001, cinnamon oil: *t_dunnett_* = −20.53, *p* < 0.001, cinnamyl alcohol: *t_dunnett_* = −17.55, *p* < 0.001, citral: *t_dunnett_* = −17.83, *p* < 0.001, citronellal: *t_dunnett_* = −21.01, *p* = 0.001, citronellol: *t_dunnett_* = −9.19, *p* = 0.006, ethyl cinnamate: *t_dunnett_* = −39.12, *p* < 0.001, eugenol: *t_dunnett_* = −19.39, *p* < 0.001, farnesol: *t_dunnett_* = −33.76, *p* < 0.001, geraniol: *t_dunnett_* = −8.98, *p* = 0.005, isoeugenol: *t_dunnett_* = −15.10, *p* < 0.001, phellandrene: *t_dunnett_* = −12.31, *p* < 0.001, star anise oil: *t_dunnett_* = −18.57, *p* < 0.001). When cinnamon oil, cinnamaldehyde, and ethyl cinnamate were offered at a concentration of 1.0%, no uptake of the test solution could be measured and low percentages of flies with blue-colored abdomen were observed (Figure 2, Table 4). Moreover, when cinnamaldehyde at 0.1%, and isoeugenol, cinnamyl alcohol, and cinnamaldehyde were consumed at a concentration of 1%, 10–16% of the tested females died within the 4 h test period. Mortality data were not statistically analyzed since few dead flies were counted in some of the treatments and replications. Results from GC–MS analyses showed that test substances remained detectable in the liquid food mix until 24–48 h after the CAFE assays.

#### 3.1.3. Oviposition Assays

**Figure 3:**
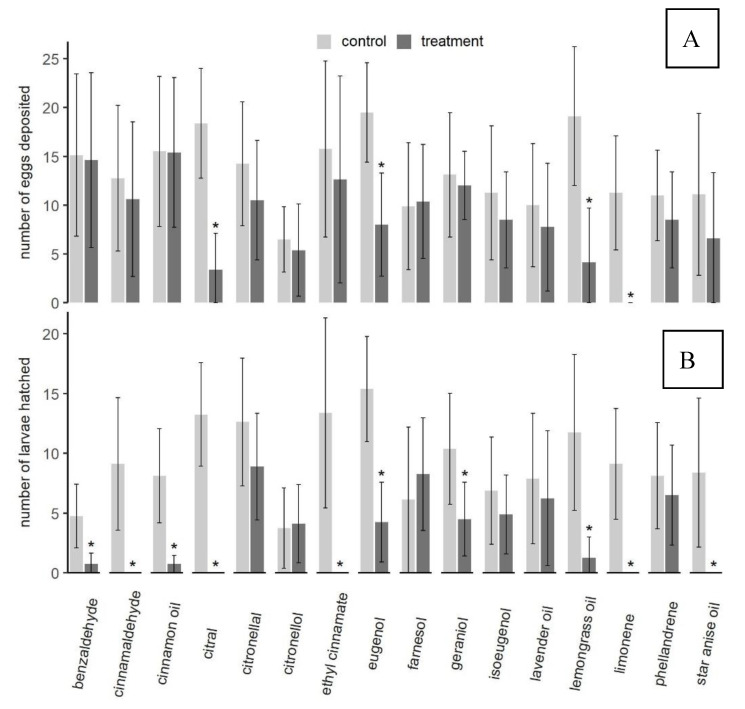
(**A**). Comparison of egg numbers deposited per each group of five females in control and test medium for each of the natural plant products tested. Oviposition media were offered for three hours. (**B**). Numbers of larvae hatched from eggs in test medium and control medium. Significant differences (*p* < 0.05) between treatment and control are marked with *. Data for oviposition and larval hatch were analyzed with a one-sided Welch test.

Egg laying in the raspberry oviposition medium was affected by the presence of test substances on a filter paper circle placed in the middle of the medium. Compared to the control, the three substances: citral (*t*_12.2_ = −6.28, *p* < 0.001), eugenol (*t*_14_ = −4.43, *p* < 0.001), and lemon grass oil (*t*_13.2_ = −4.69, *p* < 0.001) significantly reduced the number of eggs laid and limonene (*t*_7_ = −5.46, *p* = 0.001) completely inhibited oviposition (Figure 3A). No flies died during these 3 h assays. Behavioral observations of resting sites in the test arena compared fly numbers on either the oviposition medium, the glass arena or the filter paper with test compounds every 5 min for the first hour of the ovipositional period. No differences in fly numbers resting on either site or in fly behavior between control and treatment could be observed for any of the tested substances. Thus, no statistical analyses were applied to these data.

Significantly fewer larvae hatched from eggs when paper circles on the medium were treated with benzaldehyde (*t*_8.5_ = −4.04, *p* = 0.002), cinnamon oil (*t*_7.4_ = −5.21, *p* = 0.001), eugenol (*t*_13_ = −5.70, *p* < 0.001), geraniol (*t*_12.1_ = −2.98, *p* = 0.006), and lemongrass oil (*t*_8_ = −4.40, *p* = 0.001) compared to the number of larvae that hatched from eggs laid into the control medium. No larvae hatched from eggs laid in the raspberry medium when cinnamaldehyde (*t*_7_ = −4.66, *p* = 0.001), citral (*t*_7_ = −8.64, *p* < 0.001), ethyl cinnamate (*t*_7_ = −4.76, *p* = 0.001), or star anise oil (*t*_7_ = −3.80, *p* = 0.003) had been previously pipetted onto the filter paper (Figure 3B).

## 4. Discussion

The effects of natural plant compounds on SWD observed in our bioassays showed an uncorrelated pattern. The test substances differed concerning their contact toxicity, their influence on food uptake, and oviposition. Some test compounds, such as cinnamon oil and its main components cinnamaldehyde, cinnamyl alcohol, and the derivate ethyl cinnamate, showed high contact toxicity after 1 h with LC_90_ values of 2–4% for the pure compounds and an LC_90_ of 6% for cinnamon oil. The same substances significantly reduced or completely impeded feeding at the highest tested concentration of 1%. However, they did not act as oviposition deterrents. Several compounds, such as citral, lemongrass oil, eugenol, and limonene, significantly reduced or even completely inhibited oviposition, whereas the same substances showed low contact toxicity for adult flies (LC_90_ > 10%). Results confirmed a repellent effect for ovipositing females by limonene, lemongrass oil, eugenol, and citral, but not for citronellol. Moreover, citronellol, lemongrass oil, citronellal, and farnesol stimulated the uptake of liquid food in CAFE tests at concentrations of 0.01% and 0.1%. To our knowledge, this is the first study that tested the same compounds for contact toxicity as well as behavioral effects, including food uptake and oviposition. The results suggest that different underlying mechanisms were responsible for the effects deployed.

Compounds that acted as feeding deterrents might potentially be included as deterrents in a push–pull strategy while other tested substances that stimulated feeding at a low concentration might be effective in a bait mix combined with insecticides toxic at ingestion or when incorporated with feeding stimulants, such as yeast species, acetic acid bacteria, and protein mixtures [32,80,81,82,83,84]. Ovicidal effect of citral was also found for another species of Diptera, the malaria vector *Aedes aegypti* [85]. The blue-colored abdomen of flies after uptake of the test liquids showed that all substances were consumed in concentrations of 0.01 and 0.1%. At a concentration of 1.0%, however, consumption varied greatly and was significantly reduced for several compounds. The substances, that prove to be most toxic in the contact toxicity assays (cinnamon oil, cinnamaldehyde, ethyl cinnamate) were scarcely consumed in CAFE-tests at this highest test concentration.

Cinnamon extract is commercially applied in combination with onion and chili pepper against *D. suzukii* in soft fruits in Mexico (Progranic Gamma, PLM^®^ México), and showed a significant reduction in numbers of larvae found in treated raspberries [59]. The authors did not report if the observed effect is due to oviposition repellency or an increased mortality of eggs and first instar larvae.

Our contact toxicity assays revealed variable susceptibility of female and male flies. Five of the tested natural plant products caused significantly higher mortality in male flies. The reason for the observed sex related susceptibility warrants further investigation. A lower sensitivity of female flies towards particular natural plant products implies the application of higher concentrations of toxic compounds, since in efficient control strategies females in search of an oviposition host should be primarily targeted. These sex-dependent differences in mortality through contact were consistent over the entire test period only for cinnamaldehyde. Six other compounds (benzaldehyde, cinnamaldehyde, cinnamon oil, ethyl cinnamate, geraniol, and star anise oil) did not repel females from ovipositing, but reduced egg hatch compared to the control, might have been ovicidal after diffusion through the oviposition medium. Fruits treated with these substances, however, will only be marketable if the compounds chosen are harmless for human consumption and cannot be detected through taste or odor [86].

Other studies investigated natural compounds from plants against SWD in terms of fumigants or as ingestion toxicants [87]. Those laboratory experiments could not detect a clear candidate compound for long-range repellency. Volatile compounds could act via long-lasting dispensers, for example, in economically highly valuable fruits grown under tunnels such as raspberries, strawberries, and blueberries. Under field conditions, Renkema et al. [61] applied polymer flakes treated with deterrent compounds found in laboratory studies such as thymol and peppermint oil to strawberry plots. Results varied among sampling dates for oviposition and larval infestation in fruits. It is most problematic for the control of SWD, when application of commercial plant extracts cannot reduce the number of eggs and developing larvae in the fruits [58,59].

One of the advantages of the use of essential oils (EOs) in IPM is their high volatility and thus their brief persistence in the environment. Moreover, few negative side effects on beneficial and non-target organisms and low mammalian toxicity were recorded [88]. Combinations of different EOs can increase the efficiency as deterrents compared to single origin oils [89]. Currently available commercial mixtures for pest control contain EOs in concentrations below 5% and are based on few EOs, including cinnamon oil and lemon oil. In general, EOs function as growth regulators (i.e., neem oil) [88], and in soft-bodied insects or life stages the oils can penetrate membranes and wax layers of the cuticle and cause water loss, block digestive enzymes, and act as antifeedants [89] or affect the function of neurotransmitters for example acetylcholinesterase [56,90]. A number of test substances used in our contact toxicity bioassay resulted in high mortality of SWD after contact with the treated surface during the first 60 min of the test period. That effect was caused by low concentrations under 4% only for ethyl cinnamate, cinnamaldehyde, cinnamyl alcohol, and isoeugenol. Such rapid toxicity after contact or ingestion might be an indication of neurotoxic effects [48]. Further studies are necessary to detect the exact causes for the observed toxicity of the aforementioned test substances in our assays. For some EOs, a synergistic action with pyrethroid insecticides was observed [91]. Previous to the use in IPM strategies, it is thus essential to verify a potential interaction of an EO with insecticides of different modes of action to assure desired effects and avoid potential undesired antagonisms.

It needs to be considered, that not only synthetic insecticides can have negative effects on natural enemies such as, e.g., hymenopteran parasitoids, but also natural products with insecticidal effects against the target pest might simultaneously affect survival of parasitoids developing within pest pupae or eggs [92,93,94]. This is of special importance in the case of a species such as *D. suzukii* when several generations of the target pest overlap and thus all life stages of the pest insect co-occur in an orchard. As a prime endemic candidate for biological control of *D. suzukii* in the invaded areas, a pupal parasitoid, *Trichopria drosophilae* (Diapriidae), is currently being investigated in laboratory and field trials [26,28,29,30]. Recently, Trombin de Souza et al. [60] evaluated the insecticidal activity of EOs from *Piper* sp. against *D. suzukii* and for side effects against its pupal parasitoid, *T. anastrephae*. They found high toxicity against the target pest species after topical application and low mortality of the pupal parasitoid.

Besides the natural plant products studied, the methods of their application either on *D. suzukii* adults or on host fruits greatly affected results obtained and presented in the currently published studies. Our assays tested for contact toxicity of treated surfaces in order to simulate contact of the pest insect with treated leaves as happens usually under field conditions. Topical application of test substances is often used for synthetic insecticides in studies with pest insects. Under field conditions, however, this mode of exposure is not very likely since the flies are highly mobile and active mostly at dawn and dusk [95,96]. We observed, for some compounds, low toxicity with an LC_90_ > 10% through contact with treated surfaces. Such a high concentration of EOs might be phytotoxic under field conditions [97].

The ongoing worldwide research effort has not yet succeeded in identifying a specific attractant or repellent compound for SWD [98]. Thus, the high damage caused by the flies up to the total loss of a year’s harvest has led to continued application of insecticides with negative side effects on non-target arthropods and possible danger of resistance development in local populations of the target pest. Not only is *D. suzukii* polyphagous with a broad host range, there is also evidence that its preference for odor changes is based on physiological status [42,99,100], previous mating or oviposition experience [35], and fly phenology [101]. Such findings make the season-long use of attractive or repellent compounds in IPM strategies even more problematic. One of the most favorable strategies for growers is to focus on the reduction in fly numbers invading orchards early in the season when fly populations are small. This, however, poses the largest problem since current knowledge on the fly´s biology clearly tells that ripening fruits are always more attractive than any artificial odor.

At present, until compounds for monitoring, trapping, and attract–kill or push–pull strategies with a high specificity for *D. suzukii* are available, the use of insect nets for various susceptible cultures (i.e., sweet cherries) is the most promising sustainable means of control for *D. suzukii* [21,102,103]. If necessary, netting of entire fruit cultures can be combined with a restricted spray regime prior and shortly after closing the nets around the orchard. The possibility to coat exclusion netting around larger fruit plots with insecticidal substances that act via contact preferably in a specific manner against *D. suzukii*, warrants further investigation.

Despite ample research effort in the newly invaded countries, not a single, species-specific control method is applicable against SWD in the different infested cultures [104]. Knowledge of biology, physiology, and ecology of this pest insect, however, is broad and can be used to assess effectiveness of laboratory results with management strategies under field conditions. The natural compounds we identified as toxic or behavior-modifying will be further investigated for their potential use in attract-and-kill and push–pull strategies in orchards and in protected cultures.

## 5. Conclusions

Invasive pest insects are a major concern for fruit growers worldwide. Often the first means of control imply increased use of insecticides and might cause problems with resistance development and undesired side effects. Innovative pest control strategies involve the use of natural plant products. To date, however, no species-specific attractant of repellent has been identified acting against SWD. We investigated a number of natural plant compounds under laboratory conditions testing the same substances for toxicity and effects on feeding and oviposition. The results obtained with these experiments successfully indicate several substances that were effective either through contact toxicity, feeding stimulation or repellency, or as oviposition deterrents. Those natural compounds we identified as toxic or behavior-modifying will be further investigated for their potential use in attract-and-kill and push–pull strategies in orchards and in protected cultures.

## Figures and Tables

**Table 1:** List of natural plant products used in the bioassays.

Compound	Company	Purity
Benzaldehyde	Merck	>99%
Cinnamon oil	Fluka	Pure essential oil
Cinnamon alcohol	Sigma Aldrich	98%
Cinnamaldehyde	Fluka	99%
Citral	Fluka	>99%
Citronellal	Fluka	>80%
Citronellol	Fluka	>95%
Ethyl cinnamate	Merck	>98%
Eugenol	Sigma Aldrich	>99%
Farnesol	Sigma Aldrich	>95%
Geraniol	Fluka	>96%
Isoeugenol	Sigma Aldrich	Cis-trans-mix, 98%
Lavender oil	Taomed	100%
Lemongrass oil	Fluka	Pure essential oil
R (+)—limonene	Sigma Aldrich	>95%
Phellandrene	Fluka	>99%
Star anise oil	Dragoco	Pure essential oil

**Table 2:** LC_90_ of substances tested for contact toxicity against adult spotted wing drosophila (SWD). Data presented are mean values estimated from a two-parameter log-logistic model for each treatment (CI: confidence interval) and combined for male and female flies.

Test Compounds	LC_90_ after 1 h (95% CI)		LC_90_ after 4 h (95% CI)		LC_90_ after 24 h (95% CI)	
Cinnamaldehyde	3.84	(2.73–4.95)	2.92	(2.18–3.67)	2.80	(2.10–3.50)
Cinnamon alcohol	3.66	(2.59–4.73)	2.14	(0.34–3.95)	2.12	(0.92–3.32)
Cinnamon oil	5.62	(4.58–6.65)	4.26	(3.34–5.18)	3.98	(3.11–4.84)
Citral	7.54	(6.45–8.62)	8.17	(7.07–9.26)	7.64	(6.47–8.82)
Citronellol	>10.00	(10.04–14.82)	>10.00	(10.29–19.28)	>10.00	(9.95–28.49)
Ethyl cinnamate	2.68	(0.95–4.40)	<2.00	(0.00–1.34)	2.90	(1.99–3.81)
Eugenol	4.01	(3.34–4.67)	4.95	(4.05–5.86)	4.85	(3.97–5.72)
Farnesol	>10.00	(10.26–22.79)	9.47	(7.20–11.73)	6.65	(5.24–8.06)
Feraniol	7.87	(7.31–8.42)	8.28	(7.70–8.87)	8.74	(7.68–9.81)
Isoeugenol	2.05	(1.50–2.60)	1.93	(1.08–2.78)	2.05	(1.50–2.60)
Lemongrass oil	9.77	(8.26–11.27)	>10.00	(9.08–3.41)	9.46	(7.96–10.96)
Star anise oil	>10.00	(7.35–19.88)	>10.00	(0.00–99.47)	>10.00	(8.36–40.05)

**Table 3:** LC_90_ of substances compared for contact toxicity against male (♂) and female (♀) flies. Asterisks after mean concentration values symbolize significant differences between male and female flies (*p* < 0.05). The comparisons were conducted using the multcomp package. CI: confidence interval.

Test Compounds	LC_90_ after 1 h (95% CI)		LC_90_ after 4 h (95% CI)		LC_90_ after 24 h (95% CI)	
Cinnamaldehyde—♂	2.75 *	(1.65–3.85)	2.06 *	(1.54–2.57)	2.06 *	(1.56–2.56)
Cinnamaldehyde—♀	4.60	(3.22–5.98)	3.55	(2.56–4.54)	3.32	(2.40–4.25)
Cinnamyl alcohol—♂	2.77 *	(1.97–3.57)	2.13	(1.01–3.25)	2.12	(0.87–3.38)
Cinnamyl alcohol—♀	4.50	(2.85–6.15)	2.17	(0.08–4.25)	2.13	(0.93–3.32)
Cinnamon oil—♂	5.58	(4.29–6.87)	3.62 *	(2.67–4.56)	3.62	(2.67–4.56)
Cinnamon oil—♀	5.59	(4.52–6.66)	4.78	(3.66–5.90)	4.29	(3.29–5.29)
Citral—♂	6.79	(5.64–7.93)	7.59	(6.33–8.84)	6.08 *	(5.06–7.10)
Citral—♀	8.03	(6.79–9.27)	8.31	(7.23–9.38)	8.56	(7.16–9.96)
Citronellol—♂	>10.00	(9.49–14.0)	>10.00	(9.88–15.13)	>10.00	(9.56–22.32)
Citronellol—♀	>10.00	(9.82–16.17)	>10.00	(8.76–27.59)	>10.00	(6.30–42.41)
Farnesol—♂	>10.00	(9.50–17.37)	5.87 *	(4.50–7.24)	4.74 *	(3.55–5.92)
Farnesol—♀	>10.00	(7.41–33.69)	>10.00	(8.17–12.24)	7.6	(6.06–9.14)
Lemongrass oil—♂	9.41	(7.52–11.30)	>10.00	(7.89–12.24)	8.07	(6.62–9.53)
Lemongrass oil—♀	9.37	(8.27–10.47)	>10.00	(9.17–12.69)	9.55	(8.34–10.75)

**Table 4:** Five female flies were released into each of the ten vials per test substance concentration and control. Data presented are mean number of blue-colored flies (±SD) after uptake of control solution and test compound offered in three concentrations (0.01%, 0.1%, 1.0%) during the 4 h CAFE assay. The mean number of blue flies was averaged from these 10 replications/concentrations (n = 10).

Test Compounds	Control	0.01%	0.10%	1.00%
Cinnamon oil	4.3 (±1.4)	84.4 (±0.7)	63.0 (±1.8)	30.1 (±0.4)
Cinnamaldehyde	3.9 (±1.5)	84.3 (±1.6)	52.5 (±1.5)	80.4 (±0.5)
Cinnamyl alcohol	94.8 (±0.6)	73.8 (±1.1)	73.9 (±1.2)	21.3 (±0.9)
Ethyl cinnamate	4.7 (±0.7)	94.7 (±0.7)	63.4 (±1.6)	60.3 (±0.5)
Eugenol	4.3 (±0.7)	4.6 (±0.7)	3.8 (±1.2)	1.5 (±1.0)
Isoeugenol	84.3 (±0.7)	4.6 (±0.5)	4.6 (±0.5)	31.8 (±1.2)
Lemongrass oil	4.3 (±1.6)	94.8 (±0.4)	94.9 (±0.3)	42.1 (±1.6)
Limonene	4.2 (±1.9)	84.4 (±0.7)	94.8 (±0.6)	63.3 (±1.3)
Citral	3.3 (±1.0)	52.8 (±1.7)	63.3 (±1.6)	42.1 (±1.6)
Citronellal	4.8 (±0.4)	15.0 (±0.0)	94.7 (±0.9)	63.2 (±1.1)
Citronellol	3.8 (±1.6)	94.7 (±0.5)	84.4 (±1.1)	21.4 (±1.2)
Geraniol	3.8 (±1.8)	52.9 (±2.0)	73.5 (±2.0)	21.3 (±0.9)
Farnesol	4.8 (±0.4)	94.9 (±0.3)	94.7 (±0.7)	31.9 (±1.7)
Lavender oil	4.8 (±0.4)	94.7 (±0.5)	94.7 (±0.5)	84.3 (±0.7)
Phellandrene	4.0 (±1.5)	4.1 (±1.6)	84.1 (±1.6)	42.2 (±2.2)
Benzaldehyde	4.0 (±1.2)	84.2 (±0.9)	94.6 (±1.0)	63.3 (±1.1)
Star anise oil	3.5 (±1.7)	84.1 (±1.5)	15.0 (±0.0)	31.5 (±1.3)
